# Mr. James Greig Smith

**Published:** 1897-07

**Authors:** 


Mr. James Greig Smith died at his residence at Bristol, England, Friday morning, May 28, 1897, of pneumonia, aged 43 years. The shock to the professional world has seldom been so great as when it received the news of the death of this brilliant young man. He attended a consultation at Wiltshire on Monday, May 24th, and dined at a friend's house at Clifton on the evening of the same day. He then complained of feeling ill, attributing it to rheumatism, from which he almost constantly suffered. The next morning, however, symptoms of pneumonia appeared, yet no great anxiety was felt for his condition until Thursday. He then began to fail and in spite of the unremitting care of his friends Drs. Shaw and Swain, who were in constant attendance, he expired on Friday morning at 8.15. The obsequies occurred on Tuesday, June 1st, at Redland Green, which were attended by a large number of his professional brethren and friends in testimony of their respect to his memory. He was married about fifteen years ago and is survived by a widow and daughter.

James Greig Smith was born near Aberdeen in 1854, was sent to a grammar school of that city at an early age, proceeding thence to the university from which he took his degree in arts in 1873 with high honor. He took the Bachelorship of Medicine and Mastership of Surgery in 1876, in which year he was elected to the post of junior house surgeon at the Bristol Royal Infirmary, continuing his connection with that institution until his death. In 1879 he became surgeon on the staff of the infirmary and there began a surgical career that made him famous all over the world. In 1888 he began to lecture on surgery at the medical school and soon afterward published his well-known treatise on abdominal surgery, which is now going through a sixth edition and has been translated into French, German and Italian. As a writer he was clear, forceful and lucid, and as a teacher these qualities together with a perfect mastery of his subject made him the most famous of the younger exponents of abdominal surgery.

In 1894, Mr. Greig Smith delivered the address on surgery before the British Medical Association at Bristol, taking for his subject the Art of the Surgeon. This oration attained conspicuous prominence as a scholarly paper, rarely equaled in material, originality or rhetoric. It has been more quoted on both sides of the Atlantic than any similar address.

It is difficult in the brief space of a magazine obituary notice to do justice to the fame of such a man; but we may be justified in predicting that in the future wherever medicine and surgery are known and taught there will be no more conspicuous exemplar of the art, whose memory will be kept alive in amphitheatre, magazine and volume than James Greig Smith.

